# Clinical experience with remote programming of cardiac implantable electronic devices

**DOI:** 10.3389/fdgth.2025.1684695

**Published:** 2026-02-04

**Authors:** Karthik Venkatesh Prasad, Marko Tietz, Bobak Salehi, Crystal Miller, Peter Kabus, David Hayes

**Affiliations:** 1 North Mississippi Health Services Inc, Tupelo, MS, United States; 2 BIOTRONIK Inc, Lake Oswego, OR, United States; 3 BIOTRONIK SE & Co. KG, Berlin, Germany

**Keywords:** remote programming of CIEDs, cardiac implantable electronic device, remote patient care technologies, remote device programming, telemedicine, emerging medical technology, CIED innovation

## Abstract

**Background:**

As remote monitoring has become the standard of care for follow-up of cardiac implantable cardiac devices, extensions of remote monitoring can provide greater efficiency for the patient, caregiver, and industry representatives. To accomplish greater efficiency and flexibility of remote monitoring, and as an interim step toward "true" remote monitoring, LiveSupport was introduced as an innovative programmer and software solution that allows for remote programming of all current and most legacy BIOTRONIK devices.

**Objective:**

To determine the utilization and efficacy of LiveSupport as an extension of remote monitoring and the level of satisfaction with this modality. Methods Analysis of the USA dataset of LiveSupport utilization from 04/01/2022 to 04/30/2025 was completed. In addition, satisfaction with LiveSupport was assessed with a total of 159 surveys of patients, remote caregivers, and industry representative.

**Results:**

A total of 38,943 total LiveSupport sessions, from 2831 unique Renamic Neo Programmers, and 555 unique users were successfully completed. LiveSupport utilization increased substantially over time, peaking at 2,295 sessions per month. Survey results were consistent with high levels of satisfaction for patients, remote caregivers, and industry representatives. It was estimated that LiveSupport saved more than 60 min per patient in 78% of the sessions.

**Conclusion:**

LiveSupport as an extension of remote monitoring is safe and effective with marked advantages for patients, remote caregivers, and industry representatives. This technical solution is an important step toward "true" remote monitoring of cardiac electronic implantable devices.

## Introduction

Since the ﬁrst permanent pacemaker implant in 1958 (1), it was clear that regular follow-up and monitoring of cardiac implantable electronic devices (CIEDs) was critically important. CIED monitoring initially evolved from in-office follow-up to trans-telephonic monitoring at a time when pacemakers were the only CIED available (2). This practice continued for many years until the ﬁrst remote monitoring system (BIOTRONIK Home Monitoring™) was approved in the USA in 2000 (3).

The other device manufacturers followed with their version of remote monitoring which subsequently became the standard of care for patients in the United States. While all remote monitoring systems aim to connect clinicians to the patient's CIED data, the frequency and specific data fields transmitted vary. Additionally, the penetration of remote monitoring has been highly variable between continents and various countries. BIOTRONIK's remote monitoring system is unique because daily transmissions occur for any patient actively enrolled in Home Monitoring™. Although some variation in remote monitoring follow-up scheduling occurs based on the preference of the monitoring physician and/or monitoring healthcare organization, remote monitoring guidelines have been determined by multiple professional societies (4–9). Standard practice includes periodic in-person follow-up in addition to remote monitoring. In-clinic follow-up is also required if any abnormalities are noted on remote monitoring that may require device troubleshooting, optimization, and/or re-programming.

For patients with CIEDs, if a problem or actionable event is recognized by remote monitoring, the absence of timely in-clinic follow-ups, device optimization, and technical support, could lead to critical health issues, including device malfunctions, undetected arrhythmias, and potential deterioration of underlying cardiac conditions.

An effort to extend remote programming options that would allow the patient to be programmed at a healthcare site closer to their home and obviate the need to travel to their usual device clinic, led to the development of a Home Monitoring™ extension that supports remote interrogating, viewing and programming of a patient's CIED at a physician's office or healthcare facility at any distance from the location of the device expert taking responsibility for the device check and potential device programming.

## Methods

### Livesupport description

BIOTRONIK utilized the unique connectivity capabilities of their programmer, the Renamic Neo device, to develop LiveSupport (10, 11). LiveSupport allows for efficient and secure remote support for patients with BIOTRONIK CIEDs, ensuring that both patients and healthcare professionals can access technical assistance if needed. The purpose of this retrospective analysis was to demonstrate the technical feasibility and safety of interrogation and reprogramming (in the absence of complications) of this remote interrogation and programming option.

Using the Renamic Neo programmer, the clinician selects the LiveSupport tab, and a number is provided for a BIOTRONIK representative or the BIOTRONIK hotline to contact the representative responsible.

Once the clinician reads and acknowledges the Conditions for LiveSupport, providing the programmer's serial number and a nine-digit password to the representative allows a password to be generated for the LiveSupport session.

The clinician and representative will be viewing a shared programming screen in LiveView mode, in which the local clinician will maintain control of the programmer and any actions. The remote device expert can highlight certain areas or controls on the Programmer screen, but the local clinician is the only one who can perform any actions or changes on the interrogated device. If LiveControl is utilized, the company representative with programming expertise is given permission to control the interrogation and programming.

Programmer options exist to allow the clinician to take control at any time and to return to LiveView if needed. (A more detailed explanation of using this technology is provided in Supplementary Material.).

### Operationalizing LiveSupport

The Renamic Neo programmer was required for LiveSupport application. As a result, LiveSupport utilization was initially dictated by the availability of this speciﬁc programmer.

The company employee involved could utilize a variety of devices to interact with the LiveSupport request, including smartphones, tablets, or computers, independent of operating system (Apple, Android, or Windows). The type of connectivity between the remote site and BIOTRONIK support could be cellular, WLAN, or LAN.

LiveSupport was initially recommended for remote clinic follow-ups, troubleshooting, and remote MRI mode programming.

### Satisfaction surveys

Between April 1, 2025, and June 6, 2025, satisfaction surveys were collected from patients, clinicians, and BIOTRONIK representatives at 42 locations/facilities across the United States from LiveSupport activations.

Data was collected immediately following the LiveSupport session. Questions posed are listed in Box 1. A true time-study was not performed, but the BIOTRONIK employee was asked to estimate the amount of time saved using LiveSupport for each case, e.g., potential driving time saved.

Box 1 **Satisfaction survey patient survey.**

**Table:** 

1. How satisﬁed are you with the interrogation/programming of your cardiac device today? • Very satisﬁed • Satisﬁed • Not sure • Dissatisﬁed • Very dissatisﬁed • Not answered 2. Remote interrogation/programming of my cardiac device was more convenient than traveling to another location. • Strongly agree • Agree • Neither agree or disagree • Disagree • Strongly disagree 3. I’m conﬁdent that people with the necessary expertise to interrogate/program my device managed my care today. • Strongly agree • Agree • Neither agree or disagree • Disagree • Strongly disagree **Remote Caregiver:** 1. Making the LiveSupport connection was easy. • Strongly agree • Agree • Neither agree or disagree • Disagree • Strongly disagree 2. The time it took to make the connection with the appropriate BIOTRONIK personnel was efficient. • Strongly agree • Agree • Neither agree or disagree • Disagree • Strongly disagree 3. I prefer LiveSupport to having the BIOTRONIK ﬁeld representative come to the office to interrogate/program a patient(s). • Strongly agree • Agree • Neither agree or disagree • Disagree • Strongly disagree **BIOTRONIK Personnel Involved:** 1. I prefer LiveSupport to having the BIOTRONIK ﬁeld representative come to the office to interrogate/program a patient(s) • Strongly agree • Agree • Neither agree or disagree • Disagree • Strongly disagree 2. I estimate that using LiveSupport for this case(s) saved me (including potential driving time): • 5 to 15 min • 15 to 30 min • 30 to 60 min • >60 min 3. My level of satisfaction with LiveSupport: • Very satisﬁed • Satisﬁed • Not sure • Dissatisﬁed • Very dissatisﬁed • Not answered 4. The reason/nature for today's LiveSupport Appointment was: • Remote Follow-up (routine) • MRI Programming or AutoDetect/MRI Guard activation • Non-routine programming change to accommodate Provider or patient request • Lead diagnostics • Non-lead related device malfunction • Other

### Statistical analysis

Continuous variables are reported as means with standard deviation, while categorical variables are presented as frequencies with percentages. LiveSupport utilization statistical analyses were conducted using SAS 9.4 (SAS Institute, Cary, NC).

## Results

**Figure 1:**
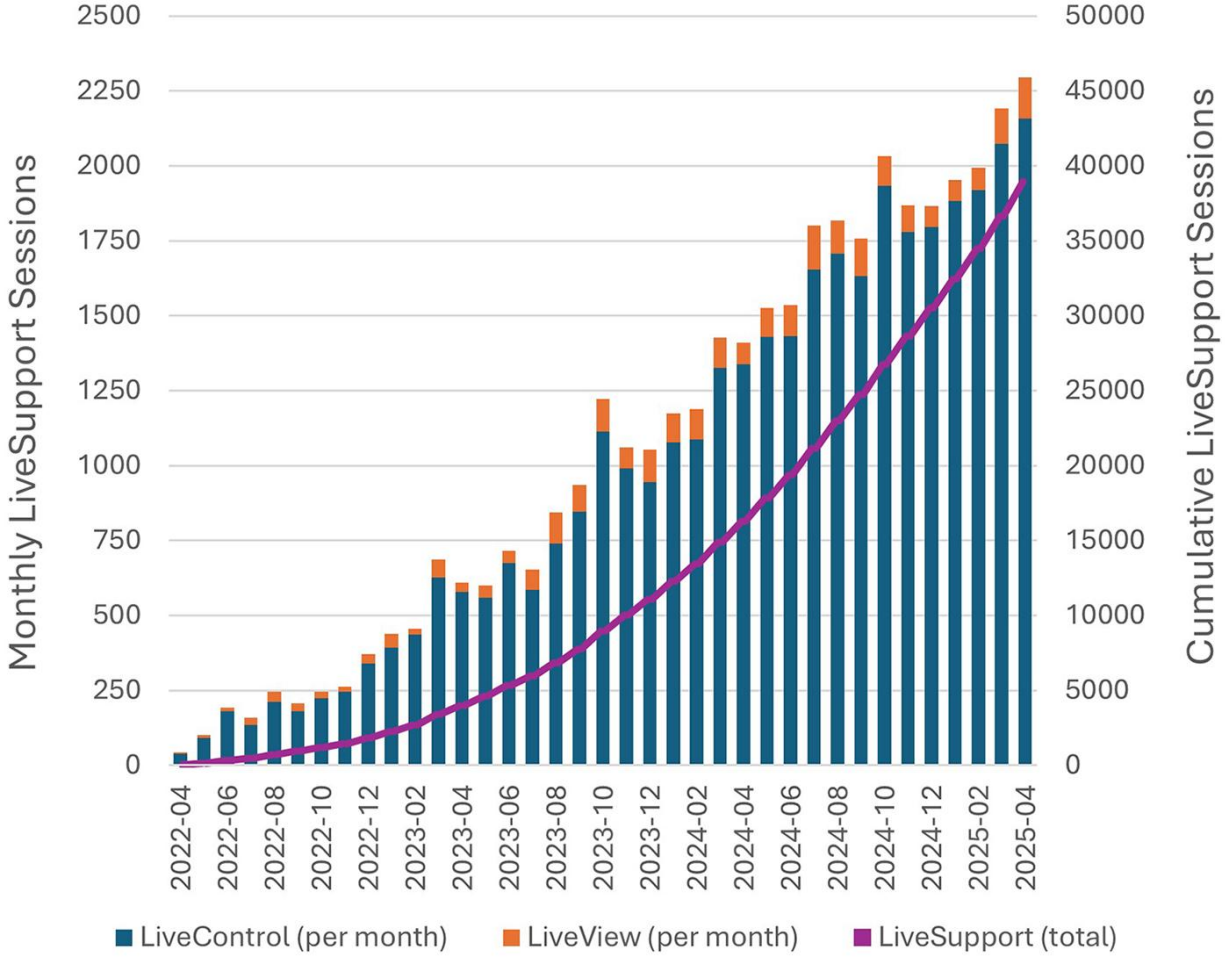
Livesupport sessions from April 2022 – April 2025.

Since LiveSupport launched on April 1, 2022 through April 30, 2025, there have been 38,943 total sessions, from 2831 unique Renamic Neo Programmer, and 555 unique users. Monthly session counts climbed over time, averaging 2,028 ± 177 sessions in the most recent 6 months (November 2024 to April 2025; Figure 1). Signiﬁcant day-to-day variability in the number of sessions was identified, ranging from 4 to 136 LiveSupport sessions per day in the last 6 months. Weekdays have consistently higher volumes, averaging 89 ± 21 sessions per day vs. 14 ± 4 sessions per day on weekends. LiveControl sessions were far more commonly used, accounting for 36,369 (93%) of the total sessions, the balance being 2,575 (7%) LiveView sessions.

**Figure 2:**
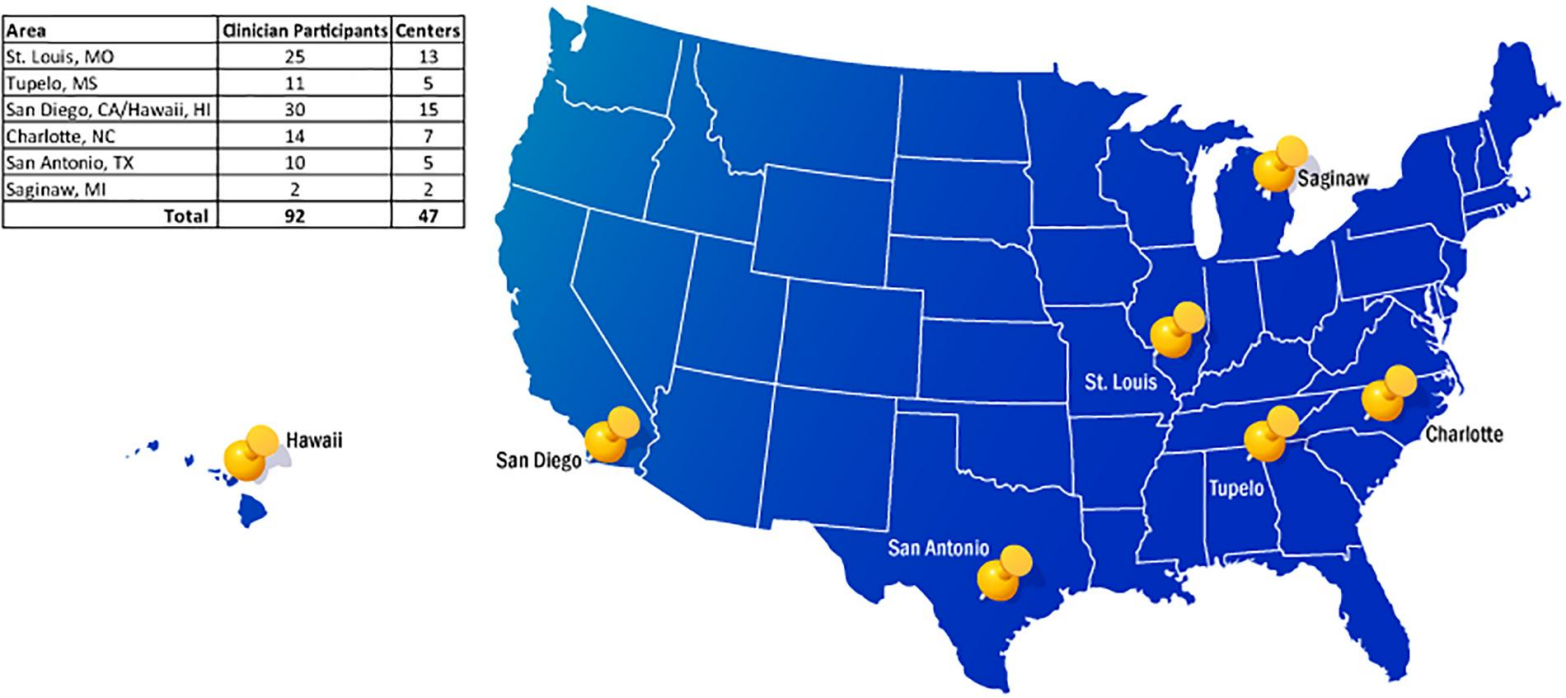
Map of participating centers in LiveSupport survey.

A total of 159 patient surveys were collected, with the help of 92 clinician participants, and 7 BIOTRONIK representatives following LiveSupport use at 47 centers (Figure 2).

**Figure 3:**
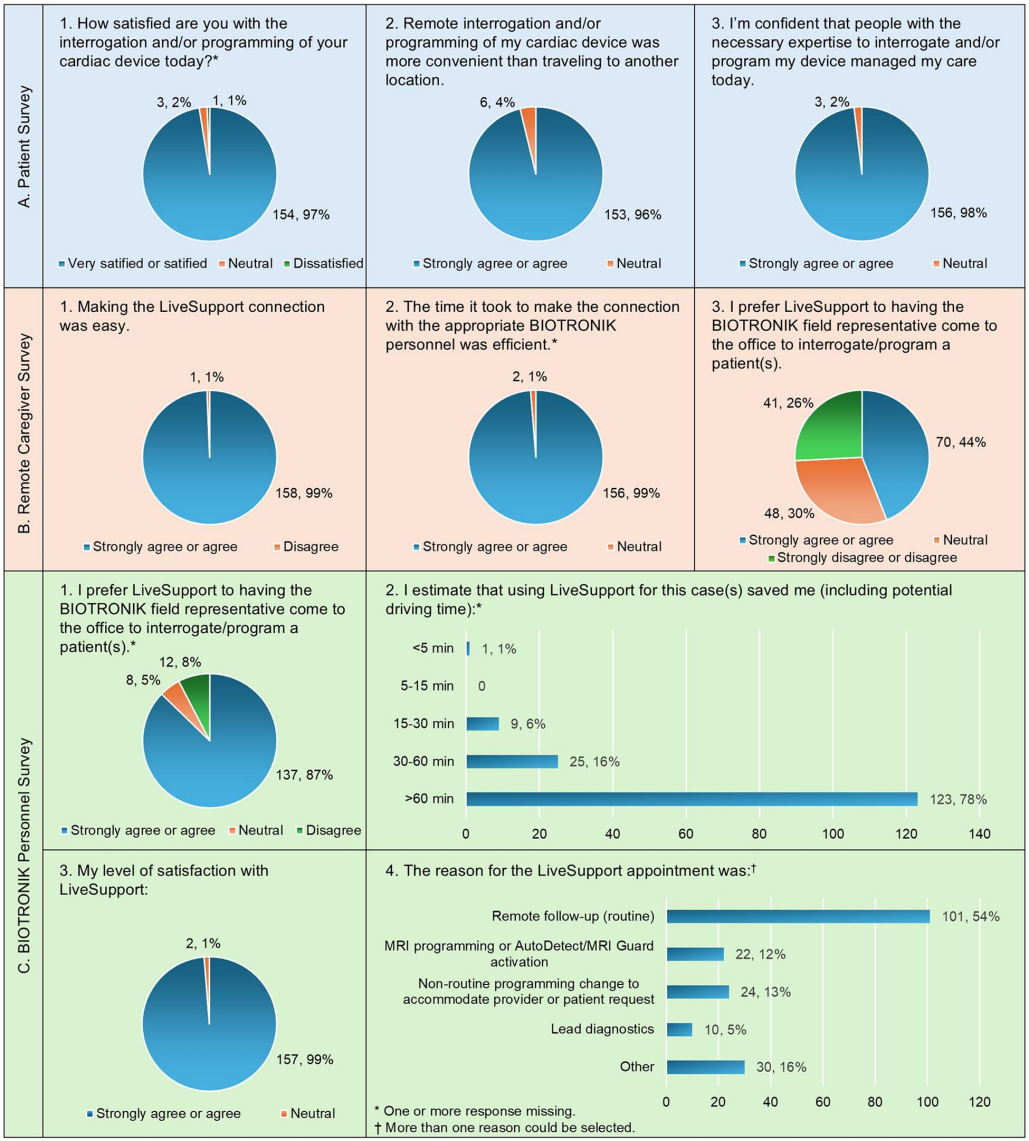
Satisfaction survey results; panel A – survey questions asked of the patient; panel B – survey questions asked of the remote caregiver; panel C – survey questions asked of the BIOTRONIK personnel performing the LiveSupport session; an estimate of “time” saved as opposed to traveling to an in-person encounter with the patient; and the reason for the LiveSupport appointment.

Satisfaction survey results are shown in Figure 3. The results were strongly positive with the majority answering highly satisﬁed/satisﬁed or strongly agree/agree for almost all questions. Not surprisingly, there was a “mixed” response when the “provider” at the remote site was asked if they preferred LiveSupport over the presence of the BIOTRONIK representative at their office.

Based on estimates of the BIOTRONIK personnel performing the satisfaction survey, they saved more than 60 min per patient in 78% of the sessions.

## Discussion

Rapid advances in digital applications are driving improved patient outcomes and clinical efficiencies in many areas of clinical medicine (12, 13). While the evidence base for standard remote monitoring of CIEDs is robust, there are limited publications regarding remote technologies that go beyond standard care. In 2013, Kloosterman et al. (14) reported on “Real-Time Remote Interrogation and Guided Reprogramming of Cardiac Implantable Electronic Devices” in a cohort of 41 patients. No complications were reported. Driven by the need during the pandemic to provide remote support to reduce COVID-19 exposure, Long et al. (15) reported a case series of 3 patients in whom remote interrogation and programming from outside of the patient's room was successfully conducted. The largest published experience to date is from Ploux et al. (16) They developed a program to remotely interrogate and program CIEDs, reporting on 115 patients at varying distances from their center from which the programming occurred. No technical or clinical complications were reported. More recently, the same group evaluated the value of remote programming in medically underserved areas (17). There are also publications regarding remote programming to facilitate MRI access (18, 19).

In addition to building on the existing limited evidence base of remote programming of CIEDs, there is increasing interest in true remote programming of CIEDs, i.e., the ability to interrogate and program a patient's CIED in any location, including those outside of a medical environment. Although there are publications regarding the potential for true remote programming (4, 20), only one publication has been identified that describes remote programming in the patient's home. Kloosterman, et al, demonstrated the safety of remote programming by having a technician go to the home of 150 patients, each of whom completed 2 remote programming sessions. No complications were noted (21).

Potential barriers for the adoption of LiveSupport include patient and clinician acceptance, technical shortcomings of the system, and lag-time in communications. This analysis demonstrates that the ability to connect with the patient at a physician's office or remote healthcare facility has been rapidly accepted with a high level of satisfaction from all involved. It is more convenient for patients, providing easier and earlier access to care, saving time and/or cost by avoiding a longer trip to their routine follow-up center, and also saving time for the device representative. LiveSupport was predominantly used with smartphones or tablets as these platforms integrate easily into the daily workﬂow of the device experts.

LiveControl, with a device representative taking control of the session, was used with much greater frequency than LiveView, i.e., providing directions while the local caregiver maintains control of the programmer.

The success of LiveSupport is evidenced by the signiﬁcant increase in use of LiveSupport sessions since initial implementation in April 2022. Use steadily increased over time, averaging over 2,000 sessions per month currently.

During the investigation period there were no adverse outcomes reported. However, reporting of some potential problems, such as connectivity interruptions, was not tracked. (More details are described in the Limitations section.).

### Limitations

This retrospective analysis of LiveSupport was focused on usability of this feature and clinician/patient satisfaction. As such, performance of LiveSupport with respect to efficiency, wait times, connection speed, session duration, and outcomes, such as if device reprogramming was required, was not available and will need to be evaluated in future studies.

There is inherent potential bias in describing use of a technology unique to a specific manufacturer, which should be considered when evaluating technologies with similar application. LiveSupport requires the availability of the Renamic Neo programmer and cannot be loaded on legacy or competitive programmer hardware which limited technical adoptability during the initial portion of this experience. Initial set-up and connectivity may also pose a challenge, especially if the IT infrastructure at the facility is highly restrictive and at facilities where cellular network connection is poor or intermittent.

While the platform is designed to be intuitive for the great majority of users, some training and familiarity with the Renamic Neo programmer could be beneficial. Additionally, a facility-specific protocol may be used to ensure consistency of experience, regardless of operator.

## Conclusions

This retrospective analysis demonstrates that BIOTRONIK's LiveSupport system is a technically feasible and safe solution for remote interrogation and programming of cardiac implantable electronic devices (CIEDs). Over three years of implementation, LiveSupport has shown rapid adoption, high satisfaction among patients, clinicians, and device representatives, and significant time savings for all stakeholders. The ability to provide secure, real-time remote support enhances patient access to timely care, reduces travel burden, and streamlines clinical workflows without compromising safety—no adverse outcomes were reported during the study period. This underscores the potential of LiveSupport to complement standard remote monitoring and in-person follow-up, paving the way for broader integration of advanced remote programming technologies in cardiac care.

## Data Availability

The original contributions presented in the study are included in the article/Supplementary Material, further inquiries can be directed to the corresponding author.
