# Supplementary material for: Clinical experience with remote programming of cardiac implantable electronic devices
Source: Front Digit Health. 2026 Feb 4;7:1684695. doi: 10.3389/fdgth.2025.1684695 (PMC12914562; doi:10.3389/fdgth.2025.1684695)
Supplement: Supplementary file 1 [file Supplementaryfile1.docx]

**Supplement A**

for

Clinical Experience with Remote Programming of Cardiac Implantable Electronic Devices

Karthik Venkatesh Prasad, MD

Marko Tietz, MS

Bobak Salehi, MBA

Crystal Miller, MS

Peter Kabus, MS

David Hayes, MD, FHRS

Corresponding Author: Bobak Salehi [bobak.salehi@biotronik.com](mailto:bobak.salehi@biotronik.com)

Disclosures

Karthik Venkatesh Prasad consults/speaks and receives honoraria from BIOTRONIK, and J&J/Medtech. He has not received any financial support in relation to this study manuscript.

Marko Tietz, Bobak Salehi, Crystal Miller, Peter Kabus, and David Hayes are employees of BIOTRONIK.


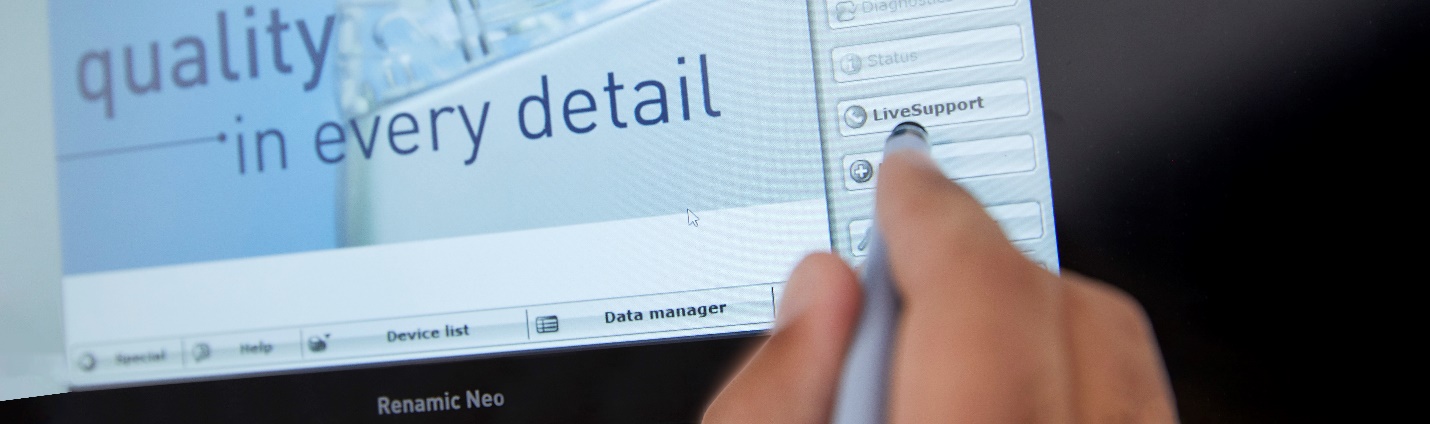


**LiveSupport**

BIOTRONIK utilized the unique connectivity capabilities of their programmer, the Renamic Neo device, to develop LiveSupport. (10,11) LiveSupport allows for efficient and secure remote support for patients with BIOTRONIK CIEDs, ensuring that both patients and healthcare professionals can access technical assistance if needed.

Using the Renamic Neo programmer, the clinician selects the LiveSupport tab, and a number is provided for a BIOTRONIK representative or the BIOTRONIK hotline to contact the responsible representative.


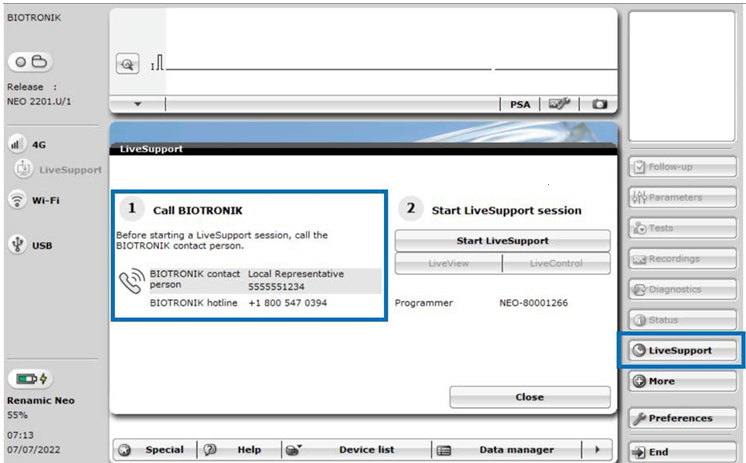


When the clinician and representative are connected by phone, the clinician selects the ‘Start LiveSupport’ button.


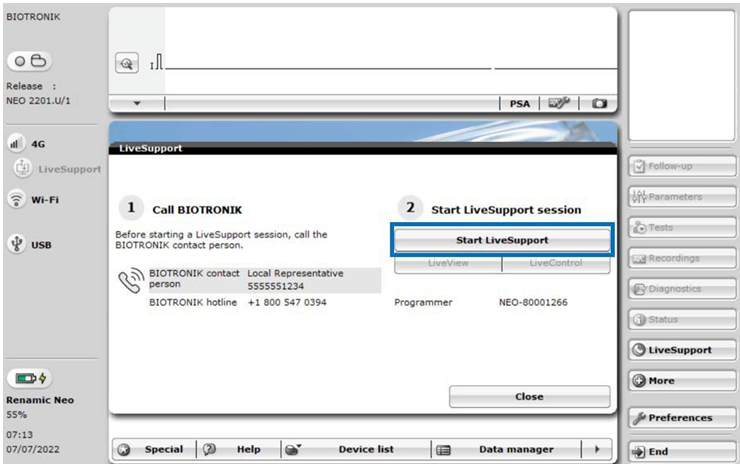


On the screen that follows, the clinician reads and acknowledges the Conditions for LiveSupport. The clinician provides the programmer's serial number and a nine-digit password to the representative. A password is generated for each LiveSupport session at the time it is initiated and remains active until the session ends. Once a session is terminated, a new password will be generated and displayed when ‘Start LiveSupport session’ is selected.

Once the programmer's serial number and password are entered, the clinician and representative will be viewing a shared programming screen in LiveView mode. In LiveView mode, the local clinician will maintain control of the programmer and any actions. The remote device expert can highlight certain areas or controls on the Programmer screen, but the local clinician is the only one who can perform any actions or changes on the interrogated device. If LiveControl is utilized, the BIOTRONIK representative with programming expertise is given permission to control the interrogation and programming. Programmer options exist to allow the clinician to take control at any time and to return to LiveView if needed. The clinician ends the session by selecting the ‘LiveSupport’ tab, followed by the ‘End LiveSupport’ button.
